# NAxtra magnetic nanoparticles for low-cost, efficient isolation of mammalian DNA and RNA

**DOI:** 10.1038/s41598-023-46868-5

**Published:** 2023-11-27

**Authors:** Eirin Johannessen Starheim, Erlend Ravlo, Jørn-Ove Schjølberg, Vanessa Solvang, Wei Wang, Nathan Robert Scrimgeour, Adeel Manaf, Sten Even Erlandsen, Per Arne Aas, Lars Hagen, Mirta Mittelstedt Leal de Sousa, Magnar Bjørås

**Affiliations:** 1 Department of Clinical and Molecular Medicine (IKOM), Norwegian University of Science and Technology (NTNU), 7491 Trondheim, Norway; 2 Department of Circulation and Medical Imaging (ISB), Norwegian University of Science and Technology (NTNU), 7491 Trondheim, Norway; 3 Department of Microbiology, Oslo University Hospital and University of Oslo, 0372 Oslo, Norway; 4 Lybe Scientific, Erling Skjalgssons Gate 1, 7030 Trondheim, Norway; 5 Proteomics and Modomics Experimental Core Facility (PROMEC) at NTNU, 7491 Trondheim, Norway; 6 Centre for Embryology and Healthy Development, University of Oslo, 0373 Oslo, Norway

**Keywords:** Nanoparticles, Epigenetics analysis, Isolation, separation and purification, Genetics research

## Abstract

A cost-effective, viral nucleic acid (NA) isolation kit based on NAxtra magnetic nanoparticles was developed at the Norwegian University of Science and Technology in response to the shortage of commercial kits for isolation of severe acute respiratory syndrome coronavirus 2 (SARS-CoV-2) RNA during the coronavirus disease 2019 (COVID-19) pandemic. This method showed comparable sensitivity to available kits at significantly reduced cost, making its application for other biological sources an intriguing prospect. Thus, based on this low-cost nucleic acid extraction technology, we developed a simple, low- and high-throughput, efficient method for isolation of high-integrity total NA, DNA and RNA from mammalian cell lines (monolayer) and organoids (3D-cultures). The extracted NA are compatible with downstream applications including (RT-)qPCR and next-generation sequencing. When automated, NA isolation can be performed in 14 min for up to 96 samples, yielding similar quantities to available kits.

## Introduction

During the COVID-19 pandemic, a shortage of commercial kits for SARS-CoV-2 RNA isolation became a limiting factor for PCR-based testing of infection status in many countries, including Norway. This prompted the development of a nucleic acid (NA) extraction technology at the Norwegian University of Science and Technology (NTNU), a technology that has since been commercialized as NAxtra by Lybe Scientific AS. NAxtra is comprised of two components, namely a customized lysis buffer and silica-coated superparamagnetic iron oxide nanoparticles^1^. The lysis buffer contains a mixture of detergent and chaotropic salt to lyse the sample and inactivate nucleases^2–4^. Upon addition of nanoparticles dispersed in isopropanol, the chaotropic agents from the lysis buffer promote binding of released NAs to the silica coating^5^. When a magnetic field is applied, the superparamagnetic properties of the nanoparticles allow isolation of adsorbed NAs from the solution^6^. Following a series of alcohol wash steps, the purified NAs are eluted in nuclease-free water and ready for use in downstream applications. In fact, NAxtra has been shown to rival the performance of commonly used viral NA extraction kits at considerably reduced cost^1^. Thus, the possibility of applying this technology to isolate NA from other biological sources is currently being explored.

DNA or RNA purification from mammalian sources is frequently required in molecular research, e.g., for disease-related studies. Although numerous methods have been developed over the years^2,6,7^, the most widely used extraction procedures include the standard phenol–chloroform method^8^, and column- or bead-based commercial kits^7^. Regarding the former, phenol and chloroform are hazardous chemicals that may contaminate the purified sample and affect downstream analyses^9^. Meanwhile, commercial kits are costly, with those relying on spin-columns being low throughput and difficult or impossible to automate. Cost-effective, high-throughput alternatives exhibiting comparable or improved performance to the current commercial kits would lower expenses for molecular research and help to accelerate advancements in these fields. For this purpose, we developed an automatable, simple, and efficient method for NA isolation from mammalian cells and organoids based on the low-cost NAxtra technology. We show that the NAxtra-based method is successful in isolating high-integrity DNA and RNA from various cell and organoid types. It has a flexible input range from one hundred to one million cells and produces similar results to commonly used commercial kits while being less costly, less time-consuming and more user friendly. We have implemented the method on the robot systems KingFisher Flex and KingFisher Duo Prime for automated extraction of 96 or 12 samples, respectively. This enables rapid and consistent total NA isolation in 14 min, and DNA or RNA purification in 27 min (+ nuclease incubation time). Finally, the extracted NA is shown to be suitable for (RT-)qPCR and has been sequenced by NGS.

## Results

### Nucleic acid extraction from various cell types and amounts

**Figure 1:**
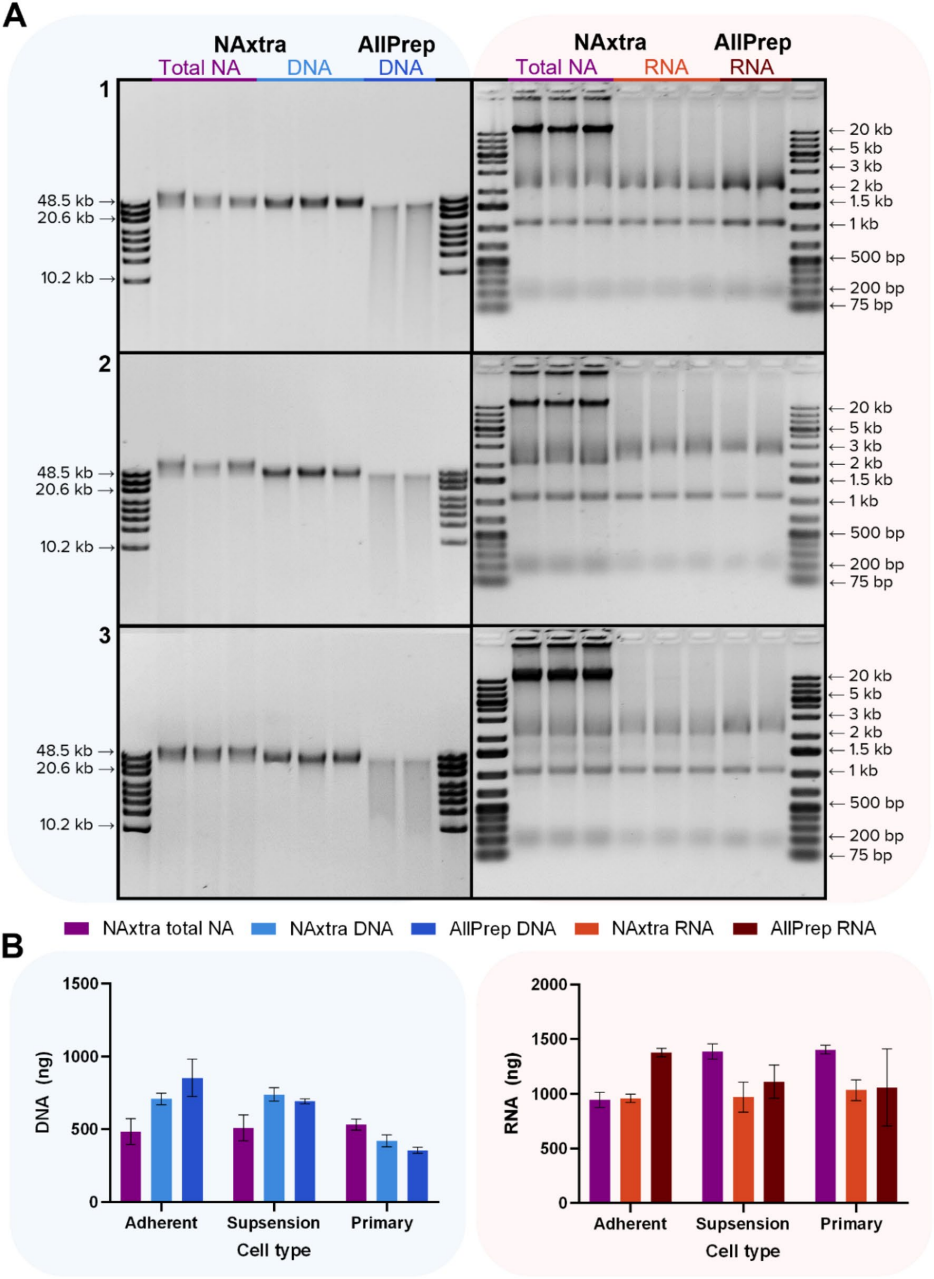
Nucleic acid (NA) extraction from adherent (HAP1), suspension (JJN-3) and primary cells (fibroblasts), comparing the NAxtra-based method on KingFisher Flex (three independent NA extractions) to the AllPrep DNA/RNA/miRNA Universal Kit (two independent NA extractions). (**A**) NA extracted from 100,000 cells of 1 = HAP1, 2 = JJN-3 and 3 = fibroblasts, separated on a 0.4% agarose gel (left; 2% eluate applied) with GeneRuler High Range DNA ladder (Thermo Scientific), or 1.2% agarose gel (right; 20% eluate applied) with GeneRuler 1 kb Plus DNA Ladder (Thermo Scientific). Gel images have been inverted and cropped; original gels are presented in Supplementary Fig. 1. (**B**) Yields (± 1 SD) of DNA (left) and RNA (right) extracted from 100,000 cells, as measured by Quant-iT RNA/DNA assay (Invitrogen).

Mammalian cell lines are widely used in molecular research as model systems for studying cellular mechanisms, e.g. in investigations of pathomechanisms of human diseases^10^. In many cases, DNA and/or RNA analyses are fundamental to reveal key molecular mechanisms. Therefore, robust and reliable extraction of high-quality DNA/RNA is essential. To illustrate the potential of the NAxtra-based method for NA extraction from different cell types, isolation of total NA, DNA and RNA was conducted for 100,000 cells of an adherent cell line (HAP1), a suspension cell line (JJN-3) and primary cells (fibroblasts, adherent). Extraction yields (Fig. 1) were compared to a widely used commercial column-based NA extraction kit, namely the AllPrep DNA/RNA/miRNA Universal Kit (QIAGEN). The results show that NAxtra is highly suitable for NA extraction from various cell types, with similar extraction yields, as well as superior DNA integrity, to that of the popular QIAGEN kit.

The NAxtra total NA procedure has a cell input limit of 100,000 cells, while the DNA and RNA protocols can be performed for at least 1,000,000 cells. To demonstrate the flexible cell input range of the NAxtra-based method, NA extraction was conducted for 100 to 1,000,000 cells of HAP1. Extraction was evaluated in terms of DNA/RNA yield for 500,000 and 1,000,000 cells, and in terms of (RT-)qPCR cycle threshold (Ct) values, for 100 to 100,000 cells. Relationships between cell numbers (log scale) and Ct values were in all cases highly linear for the NAxtra-based procedures (R^2^ ≥ 0.998). The AllPrep DNA/RNA/miRNA Universal Kit (QIAGEN) was used for comparison.

**Figure 2:**
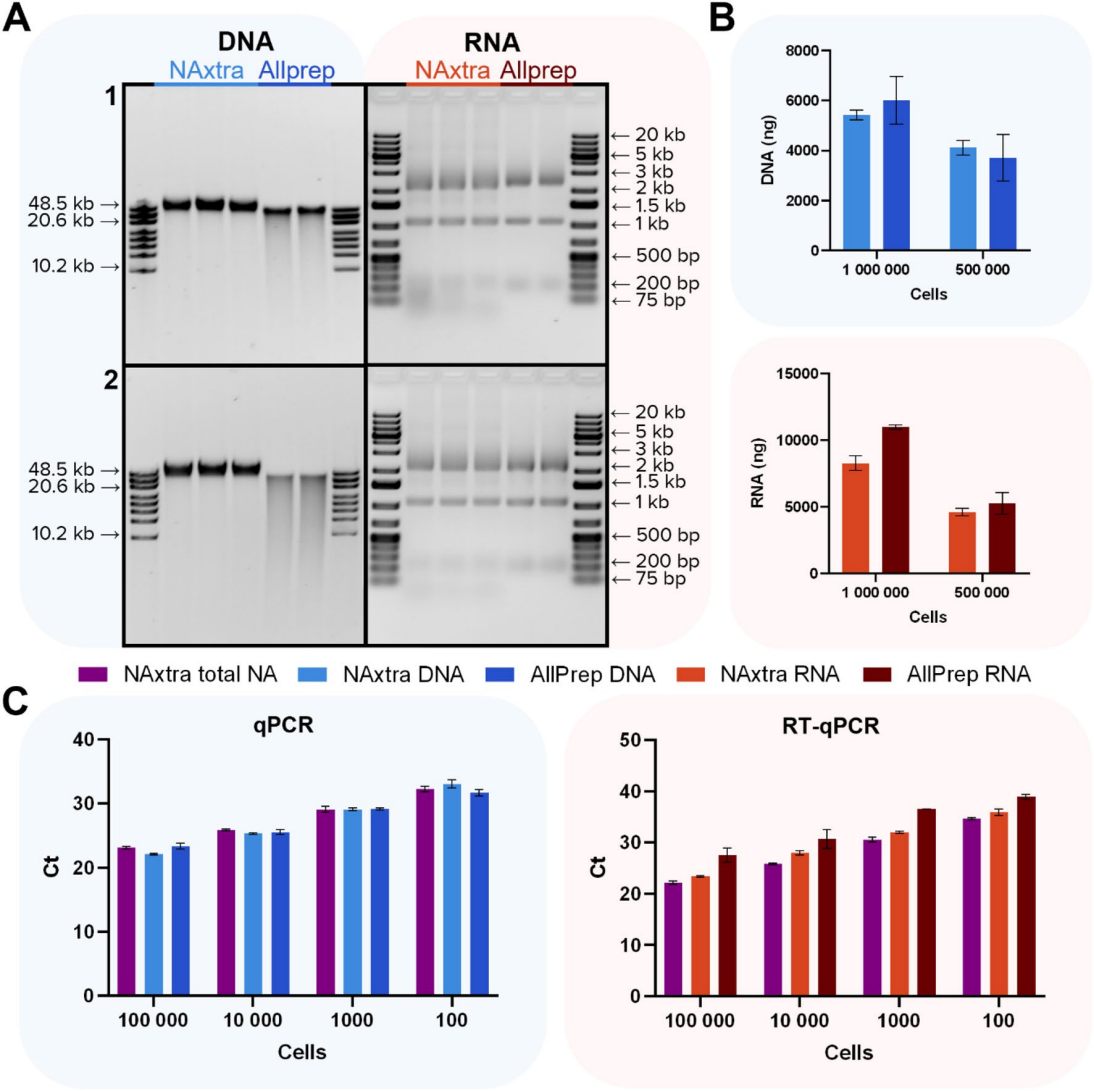
Nucleic acid (NA) extraction from 100 to 1,000,000 cells (HAP1), comparing the NAxtra-based method on KingFisher Flex (three independent NA extractions) to the AllPrep DNA/RNA/miRNA Universal Kit (two independent NA extractions). (A) NAs extracted from 1 = 1,000,000 and 2 = 500,000 cells, separated on a 0.4% agarose gel (left, 1% eluate applied for 1,000,000 cells, 2% eluate applied for 500,000 cells) with GeneRuler High Range DNA ladder (Thermo Scientific), or 1.2% agarose gel (right, 4% eluate applied for 1,000,000 cells, 8% eluate applied for 500,000 cells) with GeneRuler 1 kb Plus DNA Ladder (Thermo Scientific). Gel images have been inverted and cropped; original gels are presented in Supplementary Fig. 2. (**B**) Average NA yields (± 1 SD) from 500,000 and 1,000,000 cells, as measured by Quant-iT RNA/DNA assay (Invitrogen). (**C**) Average cycle threshold (Ct) values (± 1 SD) for DNA target (left; a 305 bp, single-locus genomic DNA amplicon from the KAPA Human Genomic DNA Quantification and QC Kit) and RNA target (right; ACTB) amplified by (RT-)qPCR of NA extracted from 100; 1000; 10,000 and 100,000 cells.

The results show comparable DNA yields for 500,000 and 1,000,000 cells between the NAxtra-based method and the AllPrep DNA/RNA/miRNA Universal Kit (QIAGEN) (Fig. 2B), with higher DNA integrity observed in NA samples extracted using the NAxtra method (Fig. 2A). Amplification of a 305 bp, single-locus genomic DNA (gDNA) amplicon (KAPA Human Genomic DNA Quantification and QC Kit) for 100 to 100,000 cells produced similar Ct values for the two kits (Fig. 2C). Overall, the two methods seem to extract comparable DNA yields, but in cases where high-integrity gDNA is required or the presence of low-integrity gDNA is undesirable, NAxtra is a favorable alternative.

For RNA extraction, the AllPrep DNA/RNA/miRNA Universal Kit (QIAGEN) produced the highest total RNA yields for 500,000 and 1,000,000 cells of HAP1 (Fig. 2B), following the trend observed for 100,000 cells of HAP1 (Fig. 1B). However, RT-qPCR detection of the mRNA of the housekeeping gene ACTB in NA extracts from 100 to 100,000 HAP1 cells was superior for NAxtra (Fig. 2C). This data suggests that, although the total RNA yield for this cell type may be reduced for NAxtra compared to the AllPrep kit, mRNA enrichment may be more efficient with the NAxtra method.

While the column-based AllPrep kits (QIAGEN) are widely used in molecular research, bead-based methods have become increasingly popular due to their practicality. To compare the efficiency of the NAxtra method to bead-based alternatives, extraction of total NA, DNA and RNA from 100,000 cells of HAP1 was performed using the MagMAX Total Nucleic Acid Isolation Kit (Applied Biosystems), MagMAX DNA Multi-Sample Kit (Invitrogen) and MagMAX *mir*Vana Total RNA isolation kit (Applied Biosystems), respectively (Supplementary Fig. 3). For DNA isolation, an average threefold reduction in yield was observed using the MagMAX DNA Multi-Sample Kit compared to NAxtra. For RNA isolation, the yields achieved were comparable between the NAxtra and the MagMAX *mir*Vana Total RNA isolation kit. For total NA extraction, the MagMAX kit required a bead-beating step, leading to a reduction in the amount of lysate recovered after bead-beating (around 56% lysate recovery per sample) for further NA extraction. Hence, the total NA yield obtained using the MagMAX kit is expected to correspond to approximately half of the NA yield obtained using NAxtra. This was observed for RNA yields in total NA samples. However, the average reduction in DNA yield for MagMAX total NA samples compared to NAxtra was more than sixfold, indicating that the NAxtra method is superior for DNA extraction from HAP1 cells (Supplementary Fig. 3). Altogether, these results indicate that the performance of the NAxtra method is comparable to the bead based MagMAX kit for RNA isolation and superior to the MagMAX kits for total NA and DNA isolation from mammalian cells.

### Nucleic acid extraction from various organoid types and numbers

**Figure 3:**
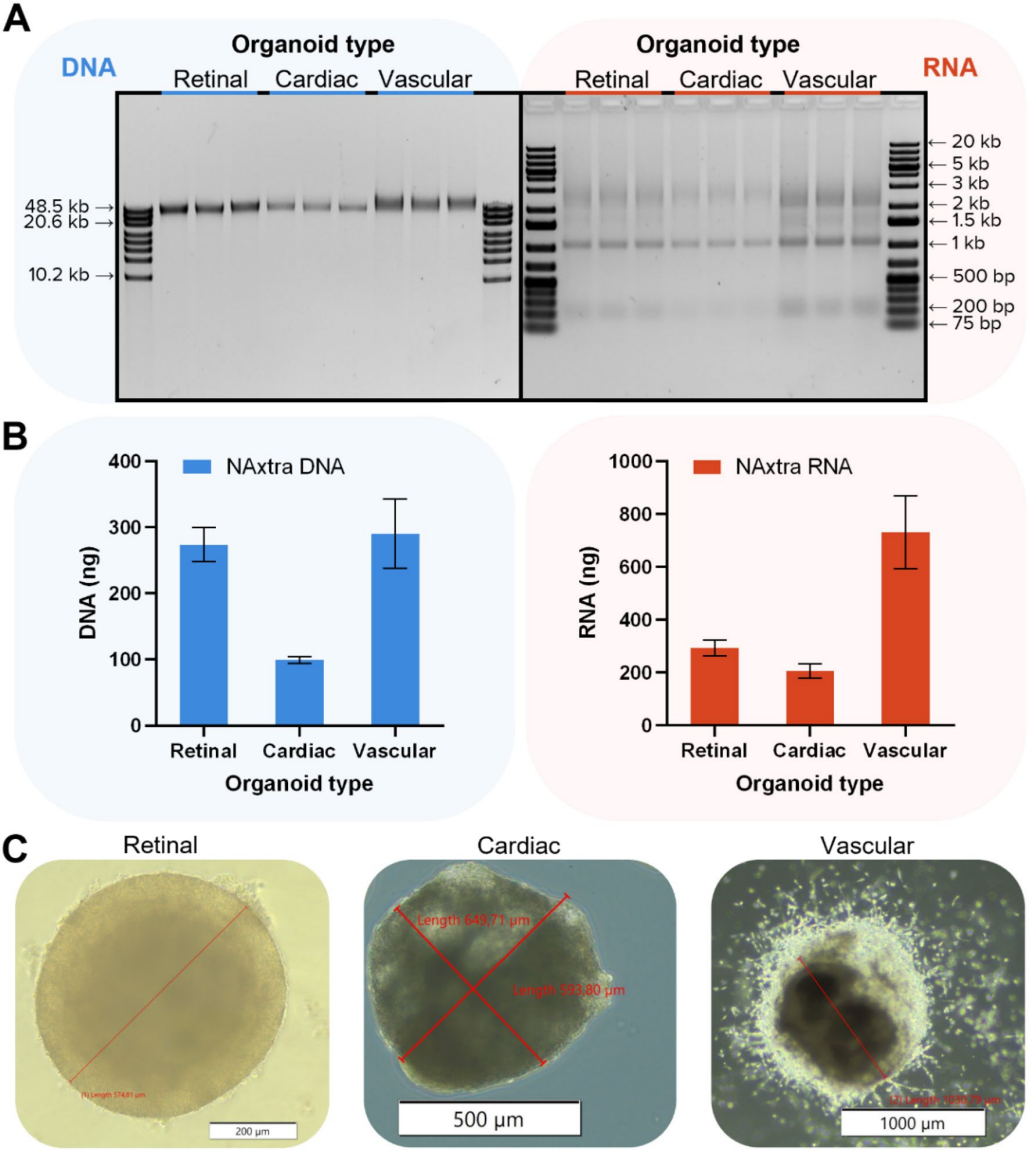
Nucleic acid extraction from triplicates of different organoid types using the NAxtra-based method on KingFisher Flex. (**A**) NAs extracted from single retinal, cardiac, and vascular organoids, with respective average diameters of 570 µm, 620 µm and 1 mm, separated on a 0.4% agarose gel (left, 5% eluate applied) with GeneRuler High Range DNA ladder (Thermo Scientific) or 1.2% agarose gel (right, 40% eluate applied) with GeneRuler 1 kb Plus DNA Ladder (Thermo Scientific). Gel images have been inverted and cropped; original gels are presented in Supplementary Fig. 5. (**B**) Average NA yield (± 1 SD), as measured by Quant-iT RNA/DNA assay (Invitrogen). (**C**) Representative images of the organoids used for NA extraction.

**Figure 4:**
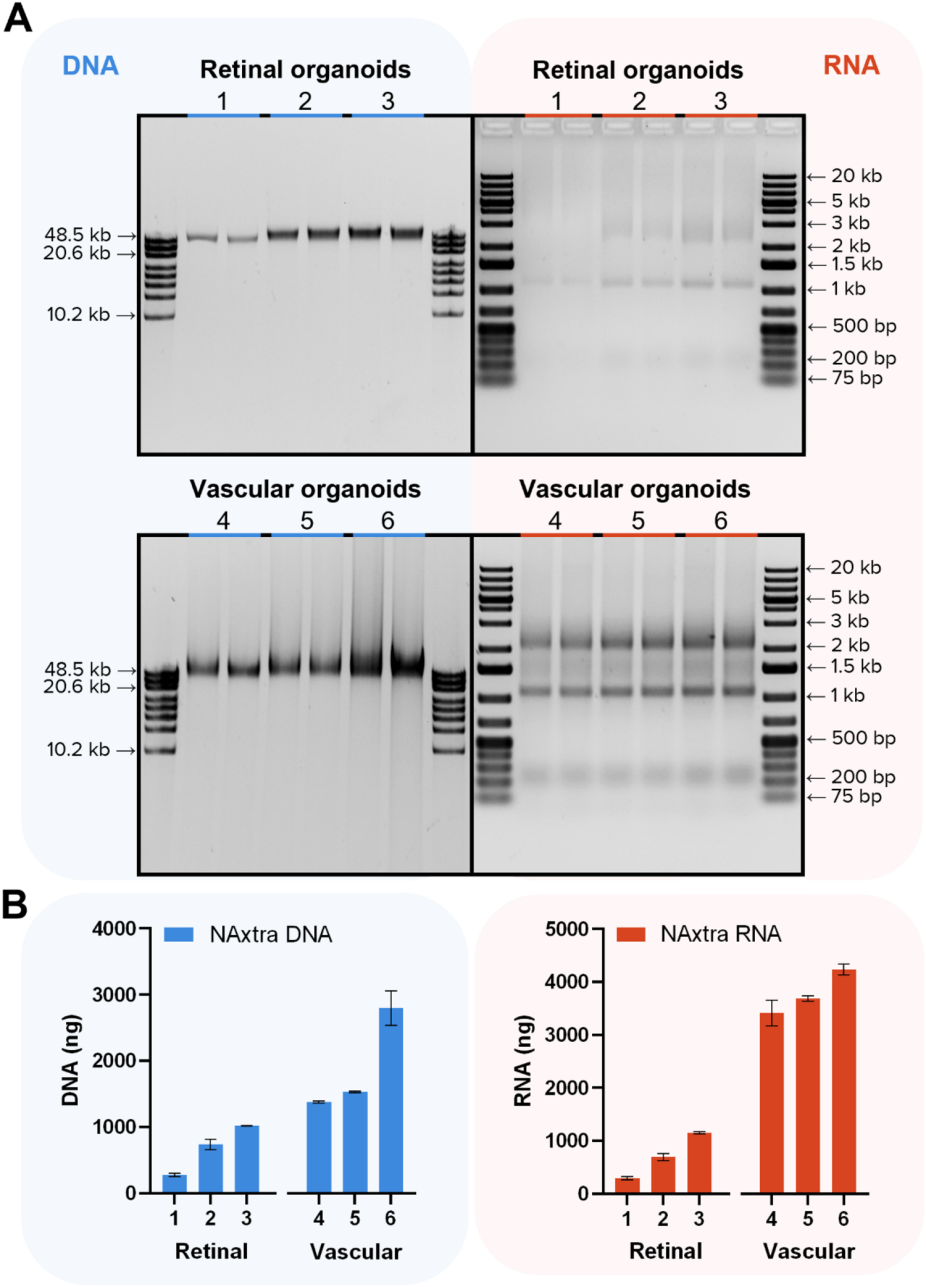
Nucleic acid extraction from duplicates of 1–3 retinal organoids and 4–6 vascular organoids using the NAxtra-based method on KingFisher Flex. (**A**) Extracted NAs separated on a 0.4% agarose gel (left, 2% eluate applied) with GeneRuler High Range DNA ladder (Thermo Scientific) or 1.2% agarose gel (right, 20% eluate applied) with GeneRuler 1 kb Plus DNA Ladder (Thermo Scientific). Gel images have been inverted and cropped; original gels are presented in Supplementary Fig. 6. (**B**) Average NA yield (± 1 SD), as measured by Quant-iT RNA/DNA assay (Invitrogen).

There are limitations associated with the use of 2D-cultured cell lines as in vitro model systems since they may exhibit different behavior to their in vivo counterparts. Model systems that resemble in vivo tissues to a higher extent are organoids, i.e. 3D organ-like cell structures originated via stem cell differentiation^11^. Compared to cell culture, the increased structural complexity of organoids may impede homogenous cell lysis during NA extraction, thereby requiring additional homogenization steps. To investigate the potential of the NAxtra procedure in extracting NAs from higher complexity samples, DNA and RNA were extracted from different organoid types (Fig. 3) and numbers (Fig. 4). DNA/RNA extractions did not require additional homogenization steps, even with increasing numbers of organoids, and were successful for all tested organoid types. The yields correlate with the organoid sizes and densities, with cardiac organoids containing fewer cells due to their hollow center.

**Figure 5:**
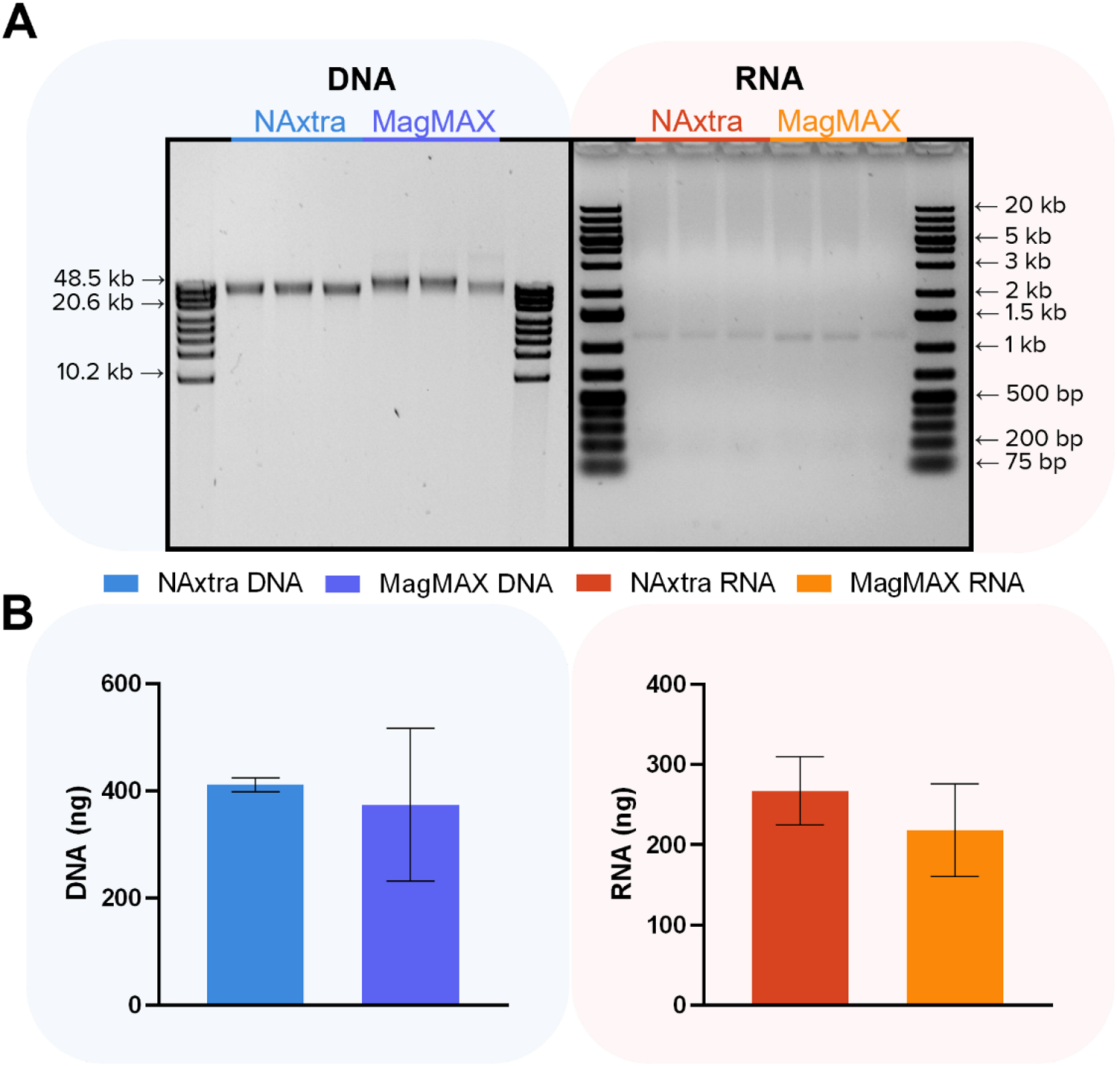
Nucleic acid extraction from triplicates of single retinal organoids, comparing the NAxtra-based method to the MagMAX DNA Multi-Sample Kit (Invitrogen) and MagMAX *mir*Vana Total RNA isolation kit (Applied Biosystems) on KingFisher Duo Prime. (**A**) Extracted NAs separated on a 0.4% agarose gel (left, 2% eluate applied) with GeneRuler High Range DNA ladder (Thermo Scientific) or 1.2% agarose gel (right, 40% eluate applied) with GeneRuler 1 kb Plus DNA Ladder (Thermo Scientific). Gel images have been inverted and cropped; original gels are presented in Supplementary Fig. 7. (**B**) Average NA yield (± 1 SD), as measured by Quant-iT RNA/DNA assay (Invitrogen).

The performance of the NAxtra method in isolating NA from organoids was also compared to the bead based MagMAX DNA Multi-Sample Kit (Invitrogen) and MagMAX *mir*Vana Total RNA isolation kit (Applied Biosystems). The resulting DNA and RNA yields from single retinal organoids were shown to be similar for NAxtra and MagMAX (Fig. 5). Lower precision in the yield measurements may be due to differences in size and density among individual organoids not necessarily reflecting differences in extraction efficiency between the methods.

### Nucleic acid integrity

Accurate assessment of DNA or RNA characteristics, e.g. (epi)genetic alterations or gene expression changes, requires NA of high integrity^12^. During NA extraction, the presence of nuclease activity or factors intrinsic to the extraction procedure, e.g., shear forces for column-based methods, may result in degradation and reduced NA integrity. To assess NA integrity, capillary or automated electrophoresis are commonly used, after which an RNA Integrity Number (RIN) or DNA Integrity Number (DIN) is determined, ranging from 1 (lowest integrity) to 10 (highest integrity)^13,14^. The integrity of DNA and RNA extracted from different cells and organoid types using NAxtra was assessed by these methods (Table 1). Our data show that the RIN and DIN values were ≥ 8.2 for all samples, and ≥ 9.5 for 72% of the samples, indicating that the extracted NA have suitable integrity for downstream applications requiring material of high quality, such as total RNA sequencing and whole genome DNA deep sequencing.

**Table 1:** Integrity analyses for nucleic acids extracted using the NAxtra-based method.

Sample type	RIN	DIN
HAP1	9.8	9.6
HAP1	9.8	9.7
HAP1	9.9	9.5
JJN-3	9.8	9.8
JJN-3	9.7	9.7
JJN-3	9.8	9.3
Primary fibroblasts	9.4	9.6
Primary fibroblasts	9.0	9.4
Primary fibroblasts	9.6	9.8
Retinal organoid	9.5	9.5
Retinal organoid	9.5	9.6
Retinal organoid	9.8	9.5
Cardiac organoid	9.8	8.9
Cardiac organoid	9.9	9.5
Cardiac organoid	9.9	9.1
Vascular organoid	8.7	9.7
Vascular organoid	8.5	9.4
Vascular organoid	8.2	9.6

RNA Integrity Number (RIN) and DNA Integrity Number (DIN) for RNA and DNA extracted from triplicates of different cells (HAP1, JJN-3, primary fibroblasts) and organoid types (cardiac, retinal, vascular).

### Suitability for downstream applications

**Figure 6:**
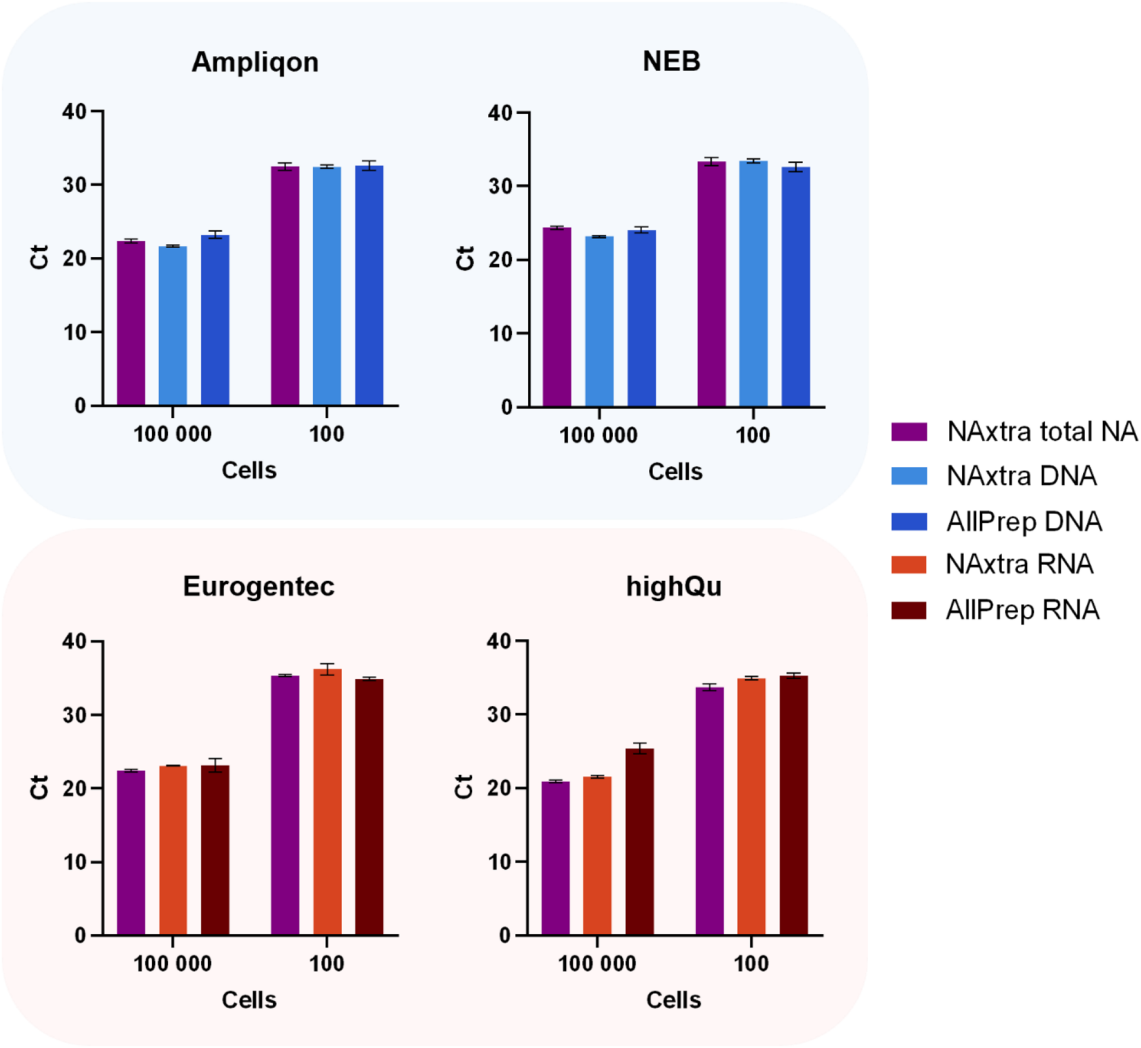
Nucleic acids (NA) extracted from adherent cells (HAP1) using the NAxtra-based method on KingFisher Flex (three independent NA extractions) compared to the AllPrep DNA/RNA/miRNA Universal Kit (two independent NA extractions). Average cycle threshold (Ct) values (± 1 SD) for a DNA target (top, MYC) and RNA target (bottom, ACTB) amplified by (RT-)qPCR of NAs extracted from 100 cells and 100,000 cells of HAP1. Two different master mixes were used for each target: RealQ Plus 2 × Master Mix for Probe (Ampliqon) and Luna Universal Probe qPCR Master Mix (NEB), or One-Step Takyon Ultra Probe 4X MasterMix (Eurogentec) and 4X 1Step RT qPCR Probe Kit (highQu).

NA extraction methods vary in their ability to remove impurities that may inhibit downstream reactions such as PCR or sequencing. Impurities can derive from solutions used in extraction, such as residual salts or organic solvents, or from insufficient removal of cellular components. Ravlo et al.^1^ recently showed that eluates obtained using NAxtra on nasopharyngeal swabs are compatible with various RT-qPCR master mixes for detection of SARS-CoV-2 RNA. In Fig. 2C, we show that gDNA and mRNA extracted from mammalian cells using NAxtra can be amplified by (RT-)qPCR in a similar manner compared to samples isolated using the AllPrep DNA/RNA/miRNA Universal Kit (QIAGEN). To examine the flexible downstream PCR compatibility of these samples, (RT-)qPCR was performed for NAs extracted from 100 and 100,000 cells using four additional master mixes (Fig. 6). Although there are some differences in relative detection between master mixes, the results show that NAs extracted from mammalian cells using NAxtra are compatible with various reaction conditions for downstream (RT-)qPCR.

Another typical downstream application for extracted NAs is NGS, which requires high quality NA material. Whole genome 5-mC and 5-hmC sequencing with the BGI platform (GEO accession ID: GSE240177) and RNA sequencing with the Illumina platform (GEO accession ID: GSE240564) have been successfully performed on NAs extracted from mammalian cells using NAxtra.

### Extraction of small RNA species

**Figure 7:**
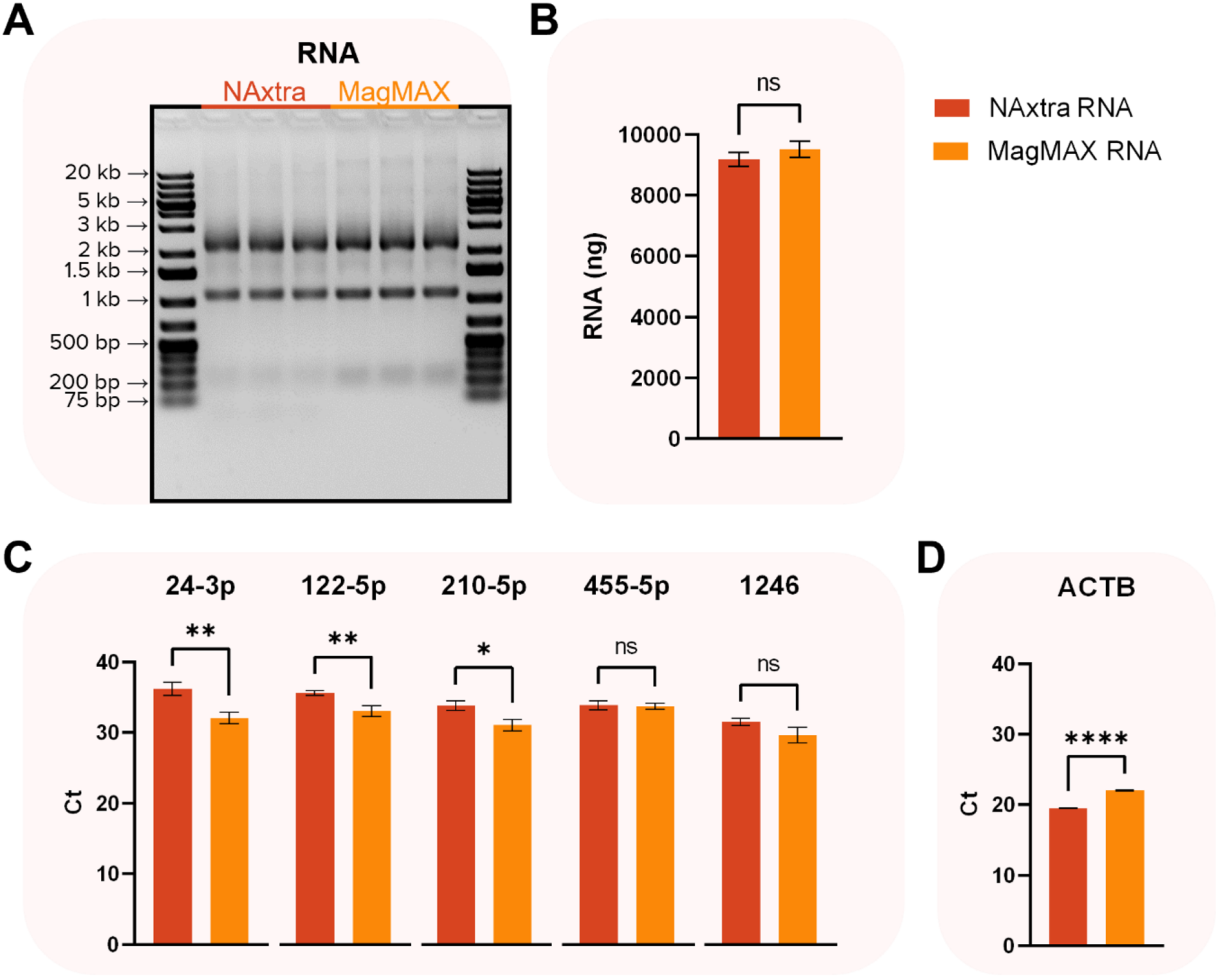
RNA isolated from triplicates of 1,000,000 cells (HAP1) using the NAxtra-based method compared to the MagMAX *mir*Vana Total RNA isolation kit (Applied Biosystems) on KingFisher Duo Prime. Statistical analysis was performed by two-sided, unpaired t-tests, in which ns (non-significant) = *P* > 0.05, * = *P* ≤ 0.05, ** = *P* ≤ 0.01, and **** *P* ≤ 0.0001. (**A**) Total RNA separated on a 1.2% agarose gel (5% eluate applied) with GeneRuler 1 kb Plus DNA Ladder (Thermo Scientific). The gel image has been inverted and cropped; the original gel is presented in Supplementary Fig. 8. (**B**) Average total RNA yield (± 1 SD), as measured by Quant-iT RNA assay (Invitrogen) (**C**) Average cycle threshold (Ct) values (± 1 SD) for five miRNA targets; hsa-miR-24-3p, hsa-miR-122-5p, hsa-miR-210-5p, hsa-miR-455-5p and hsa-miR-1246, amplified by RT-qPCR. D) Average cycle threshold (Ct) values (± 1 SD) for an mRNA target (ACTB) amplified by RT-qPCR.

Small RNAs, such as microRNAs (miRNAs), are extensively studied due to their role in RNA silencing, and potential applications in disease therapy^15,16^ or as biomarkers for various diseases^17–20^. To assess the ability of the NAxtra-based method to extract small RNAs, the method was compared to a bead-based commercial kit designed to effectively purify such molecules, namely the MagMAX *mir*Vana Total RNA isolation kit (Applied Biosystems). Total RNA was extracted from 1,000,000 cells, and the presence of five miRNA targets (hsa-miR-24-3p, hsa-miR-122-5p, hsa-miR-210-5p, hsa-miR-455-5p and hsa-miR-1246) and one mRNA target (ACTB) was detected using RT-qPCR (Fig. 7). The results indicate that NAxtra extracts similar amounts of total RNA to the MagMAX *mir*Vana Total RNA isolation kit and can be used for isolation and detection of small RNA, however, with reduced sensitivity depending on the specific target. Meanwhile, the detection of the housekeeping ACTB mRNA is superior for NAxtra compared to the MagMAX *mir*Vana Total RNA isolation kit (Fig. 7 and supplementary Table 1). This suggests that, although the NAxtra technology may be suboptimal for enrichment of small RNAs, its mRNA enrichment capability is suitable for gene expression studies.

## Discussion

The NAxtra-based method for isolation of RNA and DNA from mammalian cells and organoids is shown to be highly versatile in terms of sample type and input range. When compared to alternative commercial kits, it produces similar yields while being both less costly and faster, with a processing capability of 96 samples in 14 min when automated. The extracted NAs are shown to be of high integrity and compatible with various kits for (RT-)qPCR, and NGS. Altogether, our data shows that the NAxtra method for isolation of RNA and DNA derived from mammalian cells and organoids is straightforward, flexible, rapid, and cost-effective, being an attractive alternative to commercially available alternatives.

## Methods

### Biological material

#### Cell culture

JJN-3, HAP1 and primary fibroblasts, provided by the Department of Clinical and Molecular Medicine (IKOM) at NTNU, were cultured in RPMI-1640 (Sigma-Aldrich), IMDM (Gibco) and DMEM (Sigma-Aldrich) medium, respectively. All media were supplemented with 5% fetal bovine serum (Sigma-Aldrich), 1% l-glutamine (200 mM, Sigma-Aldrich) and 1% Penicillin (10,000 U/mL) Streptomycin (10,000 µg/mL) (Gibco). Cultures were incubated at 37 °C in a Steri-Cycle CO_2_ Incubator (Thermo Scientific) containing 5% CO_2_. Cells were harvested at 70–80% confluency. For comparison of the NAxtra-method with the MagMAX *mir*Vana Total RNA isolation kit (Applied Biosystems) for 1 million cells, the cell pellets were used directly in NA extraction. For other comparisons, cell pellets were flash frozen in liquid nitrogen and stored at − 80 °C until NA isolation.

#### Organoids

Retinal, cardiac, and vascular organoids were generated following procedures described by Yamasaki et al*.*^21^, Sun et al.^22^, and Hofbauer et al*.*^23^, respectively, with minor modifications, such as changes in medium components and/or differentiation mediators. Retinal organoids derived from two individuals were collected at day 10 of differentiation, with average diameters of 630 µm and 570 µm. The smaller retinal organoids were used for NAxtra-based NA extraction from single organoids (Fig. 3), while the larger were used in NAxtra-based extraction from two and three organoids (Fig. 4). Single retinal organoids with an average diameter of 660 µm, were collected at day 24 of differentiation for the comparison between the NAxtra method with the MagMAX DNA Multi-Sample Kit (Invitrogen) and MagMAX *mir*Vana Total RNA isolation kit (Applied Biosystems). Vascular organoids made with three variations of the final culture stage, i.e., embedded in Matrigel with MV2 medium, embedded in Matrigel with neural differentiation medium (50% DMEM/F12 medium, 50% Neurobasal medium, 2% B27 supplement, 1% N2 supplement), and in neural differentiation medium without Matrigel, were collected at day 12 of differentiation with respective average diameters of 1.5 mm, 1 mm and 800 µm. The 1 mm organoids were used for extraction from single vascular organoids, while combinations of the three sizes were used for isolation from four to six vascular organoids. Cardiac organoids were collected at day 11 of differentiation with an average diameter of 620 µm. Organoids were imaged with the Olympus UC90 attached to an Olympus CKX53 Cell Culture Microscope (Holger Hartmann AS). Collected organoids were stored at − 80 °C until NA extraction.

### Nucleic acid isolation

#### NAxtra on KingFisher systems

Samples of selected cell or organoid inputs, resuspended in 100 µl PBS, were mixed with 200 µl NAxtra LYSIS BUFFER (Lybe Scientific). Each sample was added a 400 µl mixture of either 20 µl NAxtra MAGNETIC BEADS (Lybe Scientific) in 380 µl isopropanol (cell inputs ≤ 100,000), or 60 µl in 340 µl (cell inputs > 100,000). Lysis, binding, washing, and elution was performed on the KingFisher Flex Purification System with a 96 Deep-Well Head (Thermo Scientific) or KingFisher Duo Prime Magnetic Particle Processor with a 12 Deep-Well Head (Thermo Scientific). For DNA samples, RNase treatment was conducted by adding 40 µg PureLink RNase A (Invitrogen) and nuclease-free water to a final reaction volume of 100 µl (cell input ≤ 100,000) or 200 µl (cell input > 100,000), followed by a 2 min incubation at room temperature. For RNA samples, DNase treatment for cell inputs ≤ 100,000 was performed using 2.5 µl RNase-Free DNase (QIAGEN), 10 µl Buffer RDD (QIAGEN) and nuclease-free water to a final volume of 100 µl for each sample, with room temperature incubation for 10 min. For cell inputs > 100,000, a mixture of 2.5 µl RNase-Free DNase (QIAGEN), 20 µl Buffer RDD (QIAGEN) and 27.5 µl nuclease-free water was prepared for each sample. For 5 min at room temperature eluates were incubated with 20 µl of the mixture before adding the remaining 30 µl with another 10 min of incubation. Nuclease-treated eluates were combined with 200 µl NAxtra LYSIS BUFFER (Lybe Scientific) and 400 µl (cell input ≤ 100,000) or 600 µl (cell inputs > 100,000) isopropanol before another round of mixing, washing and elution was performed on the KingFisher Flex Purification System or KingFisher Duo Prime Magnetic Particle Processor. Final elution volumes for comparison to the AllPrep DNA/RNA/miRNA Universal Kit (QIAGEN) were 50 µl in all cases except for DNA from cell inputs exceeding 100,000 cells, where 100 µl was used. When comparing to the MagMAX *mir*Vana Total RNA isolation kit (Applied Biosystems), elution volumes were 100 µl for 1 million cells and 50 µl for 100,000 cells. For comparison to MagMAX DNA Multi-Sample Kit (Invitrogen) and MagMAX Total Nucleic Acid Isolation Kit, the elution volumes were 100 µl and 50 µl, respectively.

#### AllPrep DNA/RNA/miRNA universal kit

The AllPrep DNA/RNA/miRNA Universal Kit (QIAGEN) is a widely used spin column-based method that allows simultaneous purification of DNA and RNA from cells or tissue samples. DNA and RNA extraction was performed according to manufacturer’s instructions. Elution volumes for the DNA fraction were two volumes of 100 µl if the eluate was to be used for assessment of yield, and 50 µl if the eluate was to be used for qPCR. Elution volumes for the RNA fraction were 50 µl.

#### MagMAX mirVana total RNA isolation kit

The MagMAX *mir*Vana Total RNA isolation kit (Applied Biosystems) is a leading product on the market, which enables isolation of total RNA, including microRNA, from multiple sample types using magnetic beads. Extraction of total RNA was conducted on the KingFisher Duo Prime Magnetic Particle Processor with a 12 Deep-Well Head (Thermo Scientific) according to kit instructions. Elution volumes were 100 µl for 1 million cells and 50 µl for 100,000 cells.

#### MagMAX DNA multi-sample kit

The MagMAX DNA Multi-Sample Kit (Invitrogen) is designed for rapid DNA isolation from multiple sample types, including cells and organoids, using magnetic beads. DNA extraction was executed on the KingFisher Duo Prime Magnetic Particle Processor with a 12 Deep-Well Head (Thermo Scientific) according to kit instructions, including a two-step elution with final elution volumes of 130 µl.

#### MagMAX total nucleic acid isolation kit

MagMAX Total Nucleic Acid Isolation Kit (Applied Biosystems) is used for isolation of total NA from a wide range of sample types. Isolation of total NA was performed on the KingFisher Duo Prime Magnetic Particle Processor with a 12 Deep-Well Head (Thermo Scientific) according to manufacturer’s instructions. The method involved a bead-beating step that allowed only 56.1% of the lysate to be used as input for extraction. For each cell sample, isolation was conducted by two parallel runs of 115 µl lysate with a combined elution volume of 50 µl.

### Nucleic acid quantitation by fluorescence assay

DNA and RNA quantity was assessed for duplicates of each sample and five standards using Quant-iT dsDNA HS Assay Kit (Invitrogen) and Quant-iT RNA HS Assay Kit (Invitrogen), according to manufacturer's instructions.

### Real-time quantitative PCR

All qPCR reactions were performed by thermal cycling on a CFX96 Touch Real-Time PCR Detection System (Bio-Rad) with reaction volumes of 10 µl and duplicate no template controls (NTCs) for each master mix. The primers are listed in Supplementary Table 2.

#### qPCR of extracted DNA

The 305 bp, single-locus genomic DNA amplicon of the KAPA Human Genomic DNA Quantification and QC Kit was amplified according to manufacturer’s instructions.

MYC was amplified in reactions of 2.5 µl eluate, 1X MYC TaqMAN Copy Number Assay Hs00834648_cn (Applied Biosystems), and either 1X RealQ Plus 2 × Master Mix for Probe (Ampliqon) or 1X Luna Universal Probe qPCR Master Mix (NEB), with annealing at 62°C and remaining reaction conditions according to master mix instructions.

#### Reverse transcription qPCR of extracted RNA

ACTB mRNA was reverse transcribed and amplified in reactions of 2.5 µl template RNA, 1X ACTB PrimeTime XL PCR Assay Hs.PT.39a.22214747 (Integrated DNA Technologies), and either 1X qScript XLT 1-Step RT-qPCR ToughMix (Quantabio), 1X One-Step Takyon Ultra Probe 4X MasterMix (Eurogentec), or 1X 1Step RT qPCR Probe 4X Kit (highQu) with annealing at 62 °C and remaining reaction conditions according to master mix specifications.

Reverse transcription of mature microRNA (miRNA) was performed using the miScript II RT Kit (QIAGEN) with HighSpec Buffer and 12 µl RNA template input. Duplicate dilutions of the resulting cDNA were prepared according to kit recommendations, and subsequent detection of miRNA targets by qPCR was conducted with 1 µl diluted cDNA, 300 nM target-specific primer (Supplementary Table 2), 1X miScript Universal Primer (QIAGEN) and 1X *Power* SYBR Green PCR Master Mix (Applied Biosystems). Reactions were performed with enzyme activation at 95 °C for 10 min, and amplification in 40 cycles of 95 °C for 15 s, 55 °C for 30 s and 72 °C for 30 s.

### Gel electrophoresis

#### Agarose gel electrophoresis

NAs were separated on 1.2% or 0.4% agarose gels in Tris–acetate-EDTA (TAE) buffer, with 0.5 µg GeneRuler 1 kb plus DNA ladder (Thermo Scientific) or 0.1 µg GeneRuler High Range DNA ladder (Thermo Scientific), respectively. Visualization was performed by post-staining with GelRed Nucleic Acid Gel Stain (Biotium) according to manufacturer’s instructions. Gel images were acquired using the Kodak Gel Logic 200 Imaging System. Gel images presented in the results section have been inverted and cropped. Original gel images are provided in Supplementary Figs. 1, 2, 4−8.

#### Integrity analyses

RNA integrity was assessed with the Agilent RNA 6000 Pico kit on the Agilent Bioanalyzer 2100 (Agilent Technologies) according to kit instructions. The Agilent 4200 TapeStation System with GenomicDNA ScreenTapes (Agilent Technologies) was used for DNA integrity analyses according to manufacturer’s instructions.

### Statistical analyses

Statistical analyses were performed in GraphPad Prism (version 9.5.1) by two-sided, unpaired t-tests, using *P* ≤ 0.05 as the probability level determining significance.

## Supplementary Information

Supplementary Information.

## Data Availability

The authors confirm that the data supporting the findings of this study are available within the article and its supplementary materials. The NGS data have been deposited for public access in the NIH database Gene Expression Omnibus (GEO) (https://www.ncbi.nlm.nih.gov/geo/). Accession codes are GEO Dataset: GSE240564 (for the RNA sequencing data) and GEO Dataset: GSE240177 (for the methylome sequencing data). The token of the dataset GSE240564, accessible on https://www.ncbi.nlm.nih.gov/geo/query/acc.cgi?acc=GSE240564, is qjwbsuuslvyflgt; the token to review GEO accession GSE240177, accessible on https://www.ncbi.nlm.nih.gov/geo/query/acc.cgi?acc=GSE240177, is glsxqaauzdylziv.
