# Correction: NAxtra magnetic nanoparticles for low-cost, efficient isolation of mammalian DNA and RNA

**DOI:** 10.1038/s41598-026-43139-x

**Published:** 2026-03-10

**Authors:** Eirin Johannessen Starheim, Erlend Ravlo, Jørn-Ove Schjølberg, Vanessa Solvang, Wei Wang, Nathan Robert Scrimgeour, Adeel Manaf, Sten Even Erlandsen, Per Arne Aas, Lars Hagen, Mirta Mittelstedt Leal de Sousa, Magnar Bjørås

**Affiliations:** 1 Department of Clinical and Molecular Medicine (IKOM), Norwegian University of Science and Technology (NTNU), 7491 Trondheim, Norway; 2 Department of Circulation and Medical Imaging (ISB), Norwegian University of Science and Technology (NTNU), 7491 Trondheim, Norway; 3 Department of Microbiology, Oslo University Hospital and University of Oslo, 0372 Oslo, Norway; 4 Lybe Scientific, Erling Skjalgssons Gate 1, 7030 Trondheim, Norway; 5 Proteomics and Modomics Experimental Core Facility (PROMEC) at NTNU, 7491 Trondheim, Norway; 6 Centre for Embryology and Healthy Development, University of Oslo, 0373 Oslo, Norway

Correction to: *Scientific Reports* 10.1038/s41598-023-46868-5, published online 27 November 2023

The original version of this Article contained an error in the Introduction.

“This enables rapid and consistent total NA isolation in 14 min, and DNA or RNA purification in 22 min (+ nuclease incubation time).”

now reads:

“This enables rapid and consistent total NA isolation in 14 min, and DNA or RNA purification in 27 min (+ nuclease incubation time).”

The original Article has been corrected.

