# Supplementary material for: NAxtra magnetic nanoparticles for low-cost, efficient isolation of mammalian DNA and RNA
Source: Sci Rep. 2023 Nov 27;13:20836. doi: 10.1038/s41598-023-46868-5 (PMC10682382; doi:10.1038/s41598-023-46868-5)
Supplement: Supplementary file 1 — Supplementary Information. [file 41598_2023_46868_MOESM1_ESM.pdf]

NAxtra magnetic nanoparticles for low-cost, efficient isolation of mammalian DNA and RNA

Supplementary table 1. Statistical analysis of total RNA yield, microRNA detection (hsa-miR-24-3p, hsa-miR-122-5p, hsa-miR-210-5p, hsa-miR-455-5p and hsa-miR-1246) and mRNA detection (ACTB) for RNA extracted in triplicates from 1 000 000 cells (HAP1) using NAxtra versus the MagMAX mirVana Total RNA isolation kit (Applied Biosystems).

**NAxtra vs. *mir*Vana**	**Statistical significance**	**P value**
Total RNA yield	Ns	0.1739
Detection of hsa-miR-24-3p	**	0.0043
Detection of hsa-miR-122-5p	**	0.0064
Detection of hsa-miR-210-5p	*	0.0109
Detection of hsa-miR-455-5p	Ns	0.7666
Detection of hsa-miR-1246	Ns	0.0548
Detection of ACTB mRNA	****	<0.0001

The data was analysed in GraphPad Prism (version 9.5.1) by two-sided, unpaired t-tests with $P \leq 0.05$ as the probability level determining significance. Ns (non-significant) = $P > 0.05$, * = $P \leq 0.05$, ** = $P \leq 0.01$, **** $P \leq 0.0001$.

Supplementary table 2. Primers for amplification of DNA and RNA targets using (RT)-qPCR.

Target	Primer Sequence (5'-3')	Source
MYC gDNA	P	TaqMAN Copy Number Assay Hs00834648_cn (Applied Biosystems)
305 bp gDNA amplicon	P	305 bp Primer Premix in KAPA Human Genomic DNA Quantification and QC Kit (Kapa Biosystems, Roche)
ACTB cDNA	P	PrimeTime XL PCR Assay Hs.PT.39a.22214747 (Integrated DNA Technologies)
Universal tag of cDNA produced using the miScript II RT Kit (QIAGEN)	P	Universal Primer in the miScript SYBR Green PCR Kit (QIAGEN)
hsa-miR-24-3p	TGGCTCAGTTCAGCAGGAACAG	IH
hsa-miR-122-5p	TGGAGTGTGACAATGGTGTTTG	miScript Primer Assay (QIAGEN)
hsa-miR-210-5p	AGCCCCTGCCCACCGCACACTG	IH
hsa-miR-455-5p	TATGTGCCTTTGGACTACATCG	IH
hsa-miR-1246	AATGGATTTTTGGAGCAGG	IH

gDNA = genomic DNA; cDNA = complementary DNA; P = Proprietary; IH = Designed in-house

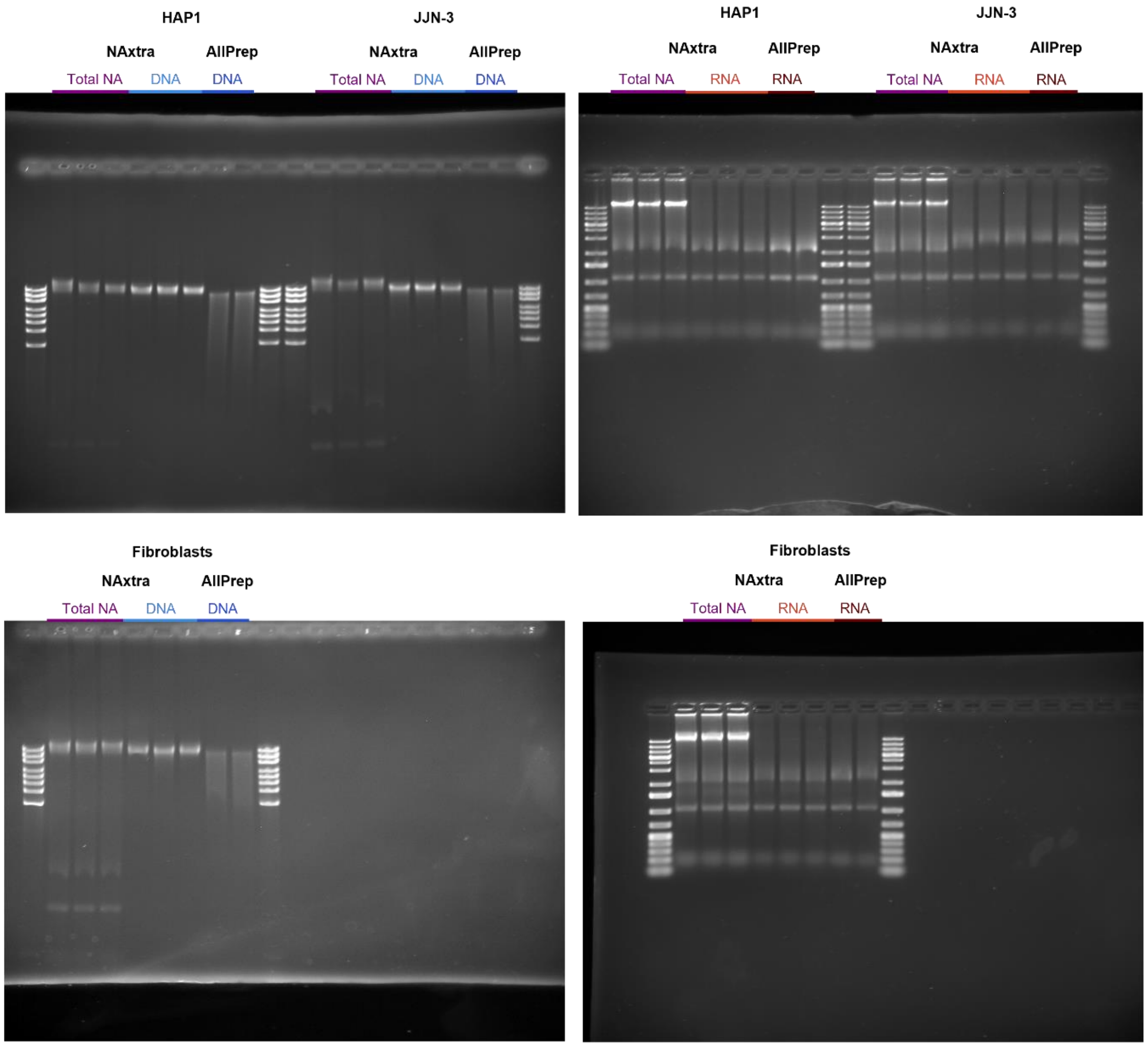

Supplementary figure 1. Original gel images for **Figure 1A**. Nucleic acid (NA) extraction from adherent (HAP1), suspension (JJN-3) and primary cells (fibroblasts), comparing the NAxtra-based method on KingFisher Flex (three independent NA extractions) to the AllPrep DNA/RNA/miRNA Universal Kit (two independent NA extractions). NA extracted from 100 000 cells of HAP1, JJN-3 and fibroblasts, separated on a 0.4% agarose gel (left; 2% eluate applied) with GeneRuler High Range DNA ladder (Thermo Scientific), or 1.2% agarose gel (right; 20% eluate applied) with GeneRuler 1 kb Plus DNA Ladder (Thermo Scientific).

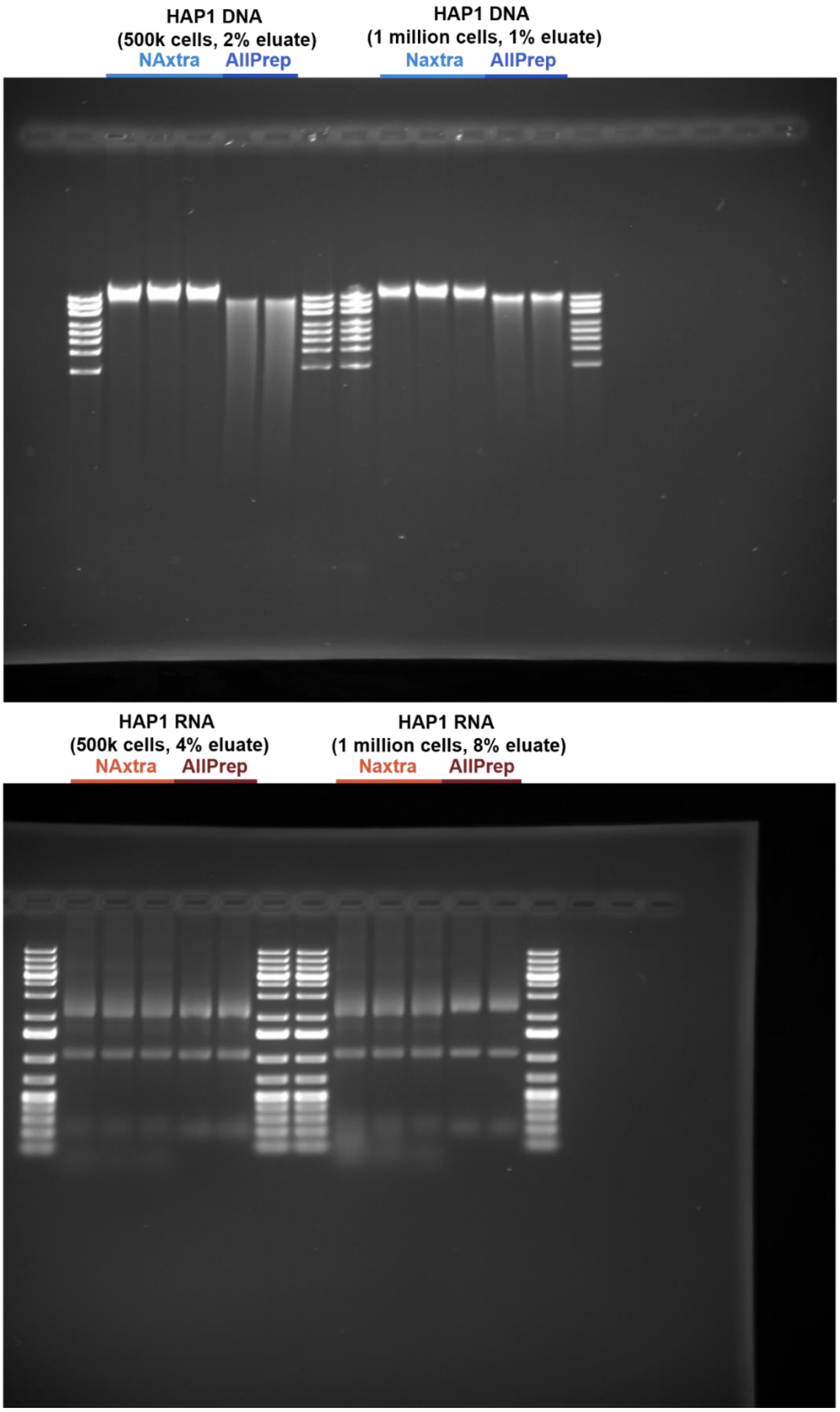

Supplementary figure 2. Original gel images for **Figure 2A**. Nucleic acid (NA) extraction from 100 to 1 000 000 cells (HAP1), comparing the NAxtra-based method on KingFisher Flex (three independent NA extractions) to the AllPrep DNA/RNA/miRNA Universal Kit (two independent NA extractions). NAs extracted from 1 000 000 and 500 000 cells, separated on a 0.4% agarose gel (top, 1% eluate applied for 1 000 000 cells, 2% eluate applied for 500 000 cells) with GeneRuler High Range DNA ladder (Thermo Scientific), or 1.2% agarose gel (bottom, 4% eluate applied for 1 000 000 cells, 8% eluate applied for 500 000 cells) with GeneRuler 1 kb Plus DNA Ladder (Thermo Scientific).

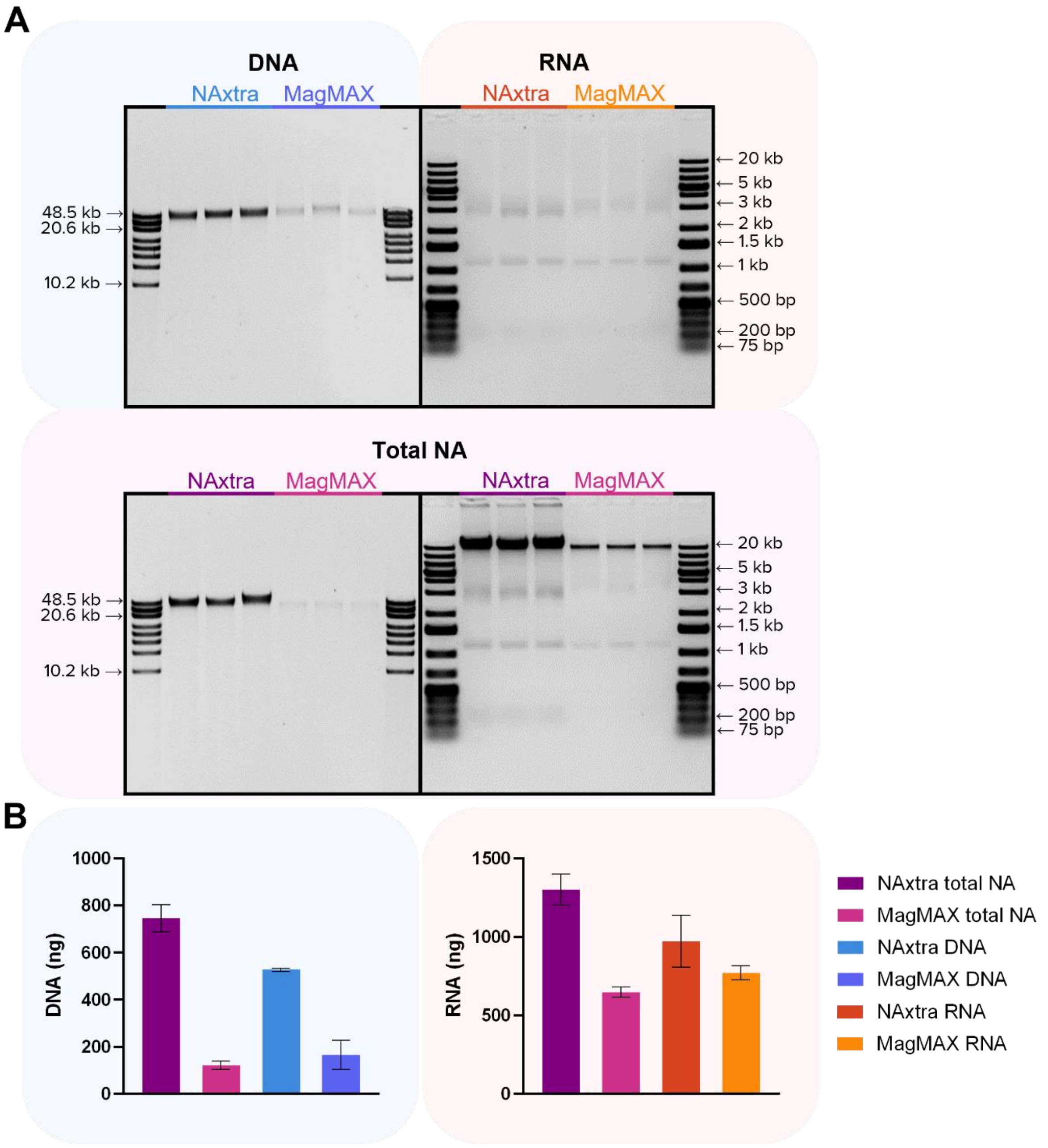

Supplementary figure 3. Nucleic acid (NA) extraction from triplicates of 100 000 adherent cells (HAP1) on KingFisher Duo, comparing the NAxtra-based method to the MagMAX Total Nucleic Acid Isolation Kit (Applied Biosystems), MagMAX DNA Multi-Sample Kit (Invitrogen) and MagMAX mirVana Total RNA isolation kit (Applied Biosystems). A) Extracted NA separated on a 0.4% agarose gel (left; 2% eluate applied) with GeneRuler High Range DNA ladder (Thermo Scientific), or 1.2% agarose gel (right; 20% eluate applied) with GeneRuler 1 kb Plus DNA Ladder (Thermo Scientific). Gel images have been inverted and cropped; original gels are presented in **Supplementary figure 4**. B) Yields (±1 SD) of DNA (left) and RNA (right), as measured by Quant-iT RNA/DNA assay (Invitrogen).

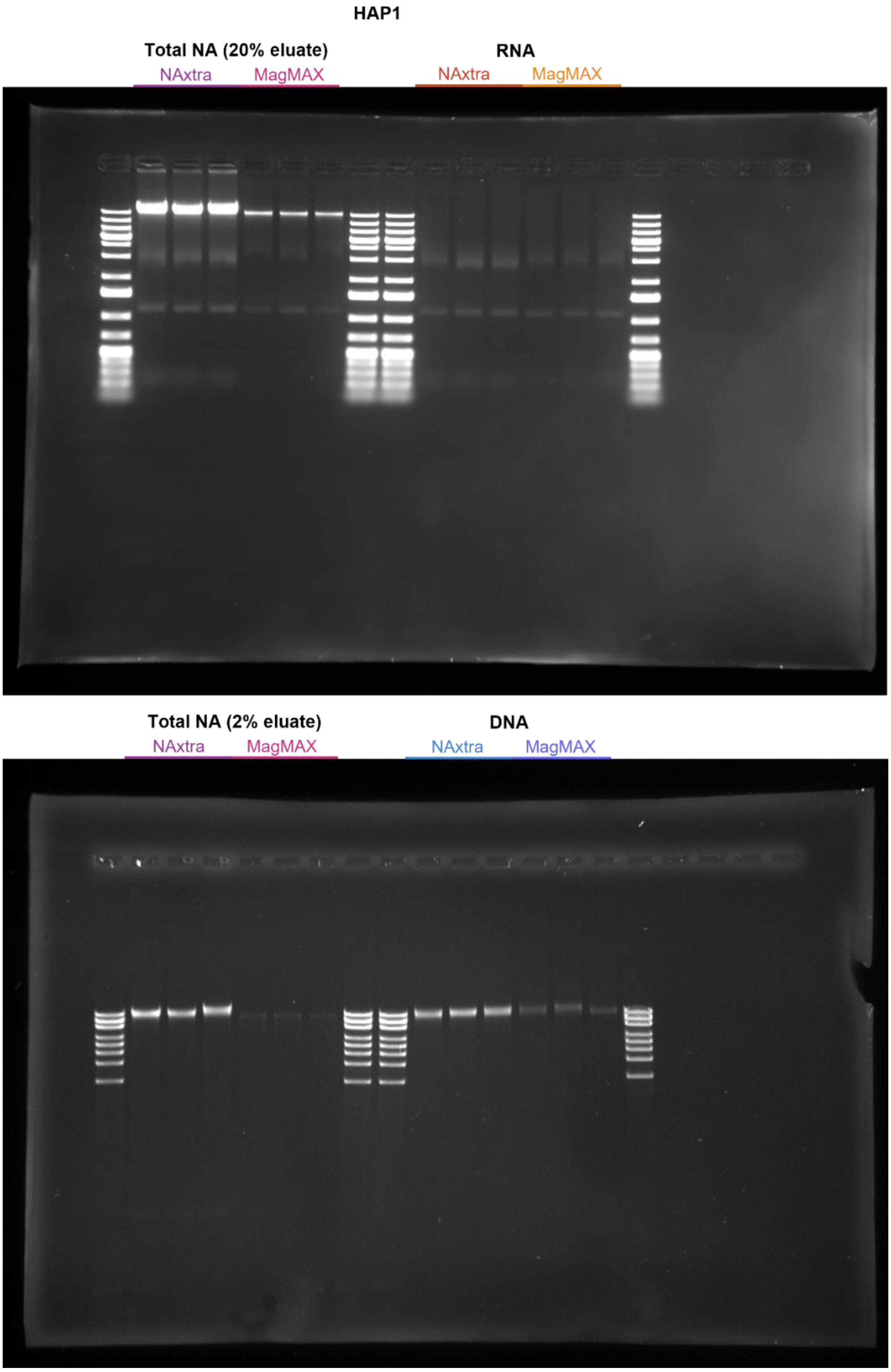

Supplementary figure 4. Original gel images for **Supplementary figure 3A**. Nucleic acid (NA) extraction from triplicates of 100 000 adherent cells (HAP1) on KingFisher Duo, comparing the NAxtra-based method to the MagMAX Total Nucleic Acid Isolation Kit (Applied Biosystems), MagMAX DNA Multi-Sample Kit (Invitrogen) and MagMAX mirVana Total RNA isolation kit (Applied Biosystems). Extracted NA separated on a 1.2% agarose gel (top; 20% eluate applied) with GeneRuler 1 kb Plus DNA Ladder (Thermo Scientific) or 0.4% agarose gel (bottom; 2% eluate applied) with GeneRuler High Range DNA ladder (Thermo Scientific).

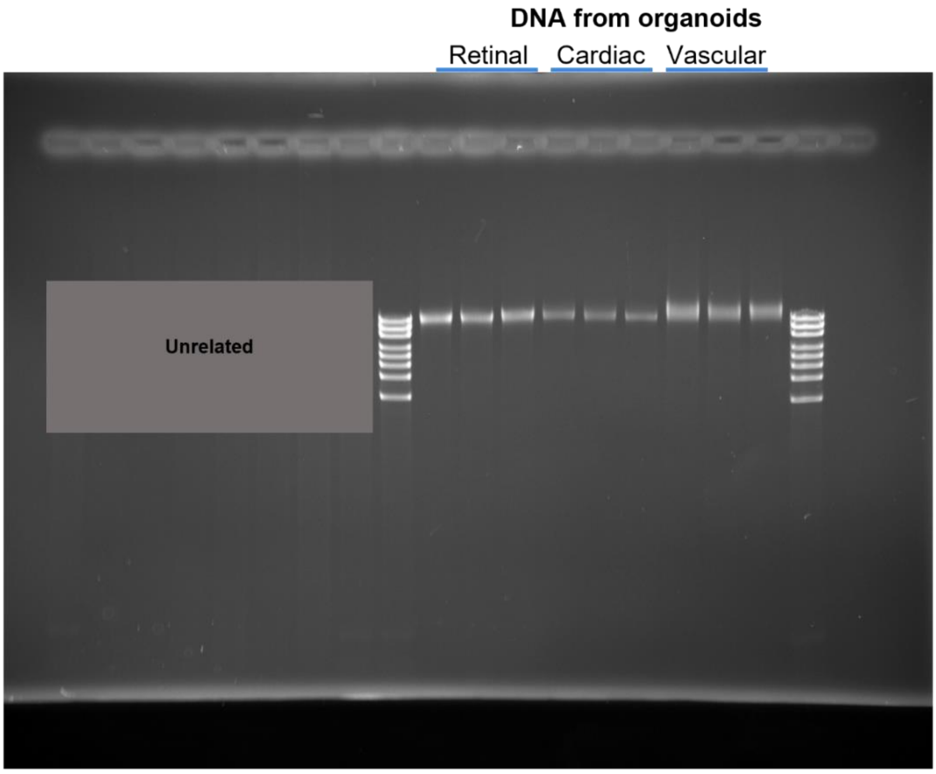

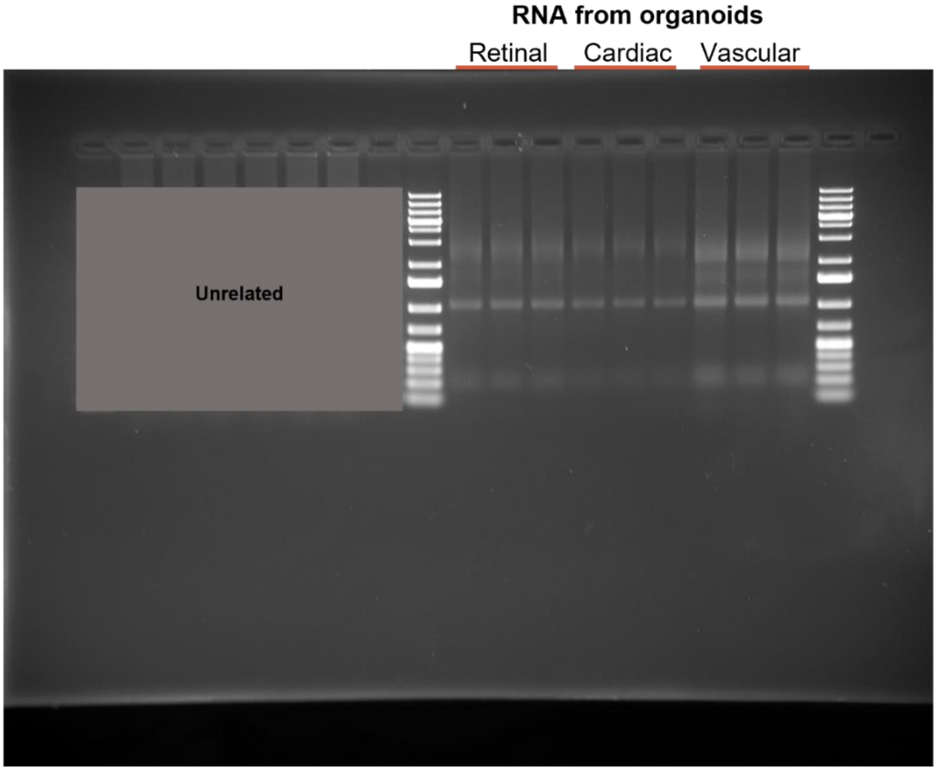

Supplementary figure 5. Original gel images for **Figure 3A**. Nucleic acid (NA) extraction from triplicates of different organoid types using the NAxtra-based method on KingFisher Flex. NAs extracted from single retinal, cardiac, and vascular organoids, with respective average diameters of 570 µm, 620 µm and 1 mm, separated on a 0.4% agarose gel (top, 5% eluate applied) with GeneRuler High Range DNA ladder (Thermo Scientific) or 1.2% agarose gel (bottom, 40% eluate applied) with GeneRuler 1 kb Plus DNA Ladder (Thermo Scientific).

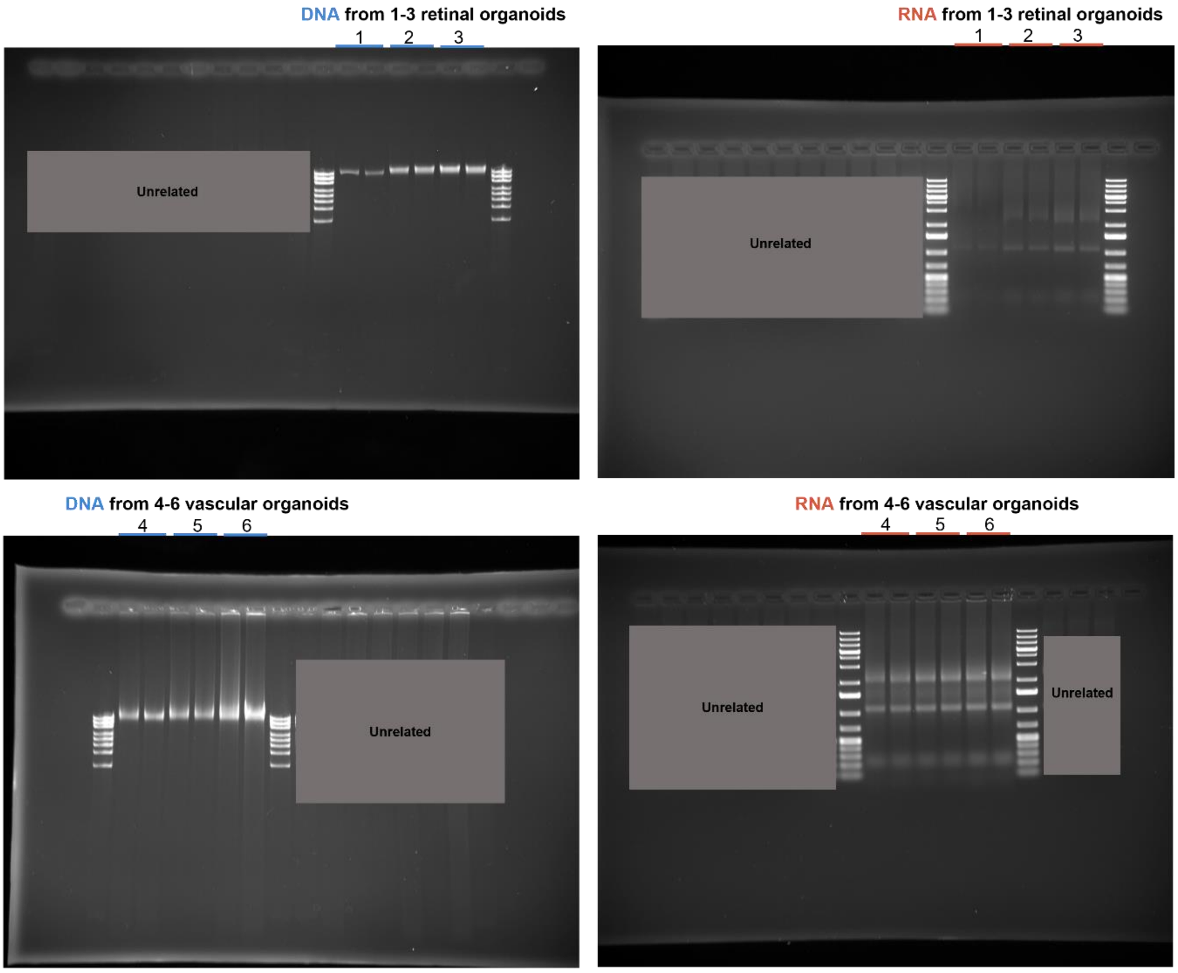

Supplementary figure 6. Original gel images for **Figure 4A**. Nucleic acid (NA) extraction from duplicates of 1-3 retinal organoids and 4-6 vascular organoids using the NAxtra-based method on KingFisher Flex. Extracted NAs separated on a 0.4% agarose gel (left, 2% eluate applied) with GeneRuler High Range DNA ladder (Thermo Scientific) or 1.2% agarose gel (right, 20% eluate applied) with GeneRuler 1 kb Plus DNA Ladder (Thermo Scientific).

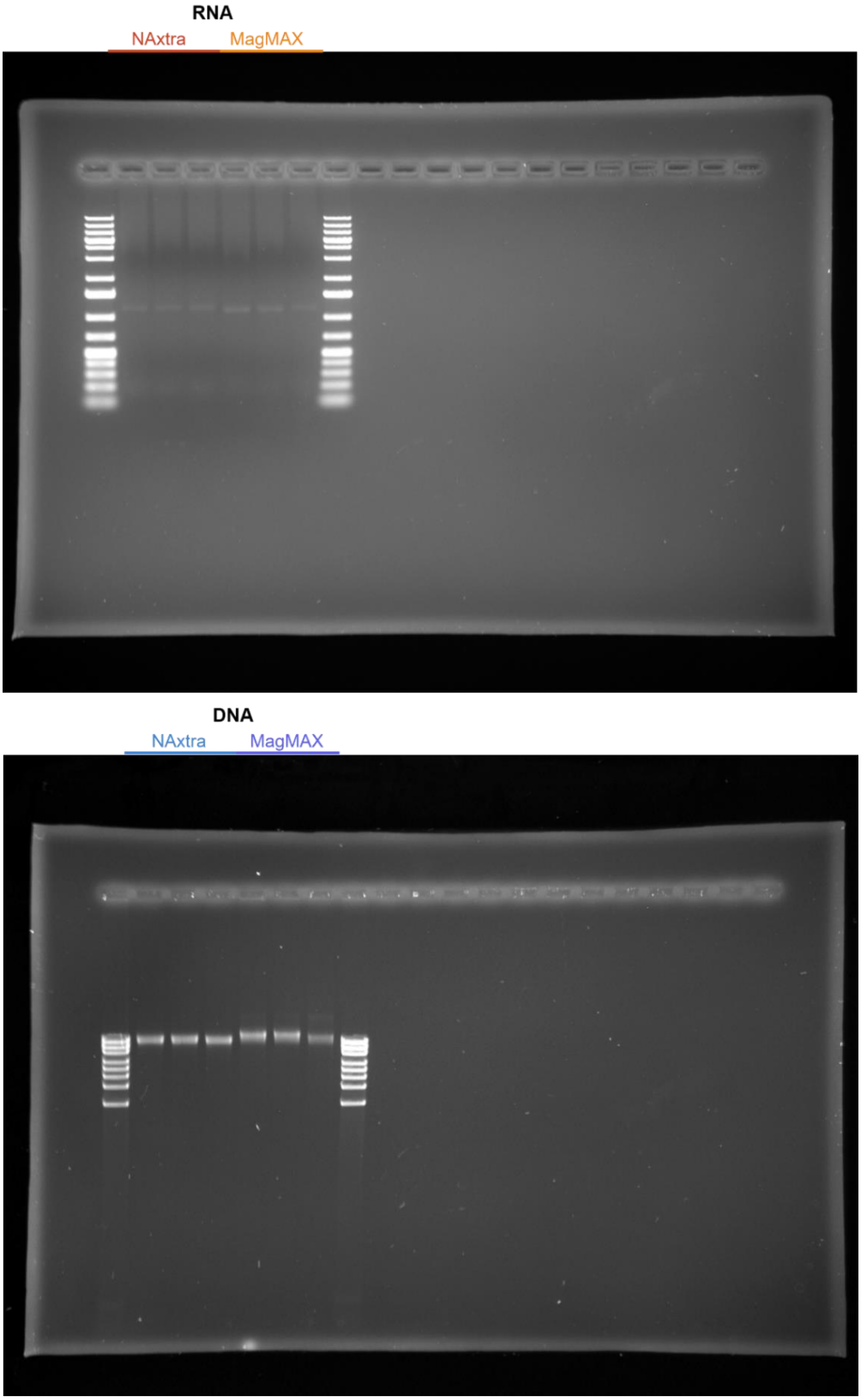

Supplementary figure 7. Original gel images for **Figure 5A**. Nucleic acid (NA) extraction from triplicates of single retinal organoids, comparing the NAxtra-based method to the MagMAX DNA Multi-Sample Kit (Invitrogen) and MagMAX *mir*Vana Total RNA isolation kit (Applied Biosystems) on KingFisher Duo Prime. Extracted NAs separated on a 1.2% agarose gel (top, 40% eluate applied) with GeneRuler 1 kb Plus DNA Ladder (Thermo Scientific) or 0.4% agarose gel (bottom, 2% eluate applied) with GeneRuler High Range DNA ladder (Thermo Scientific).

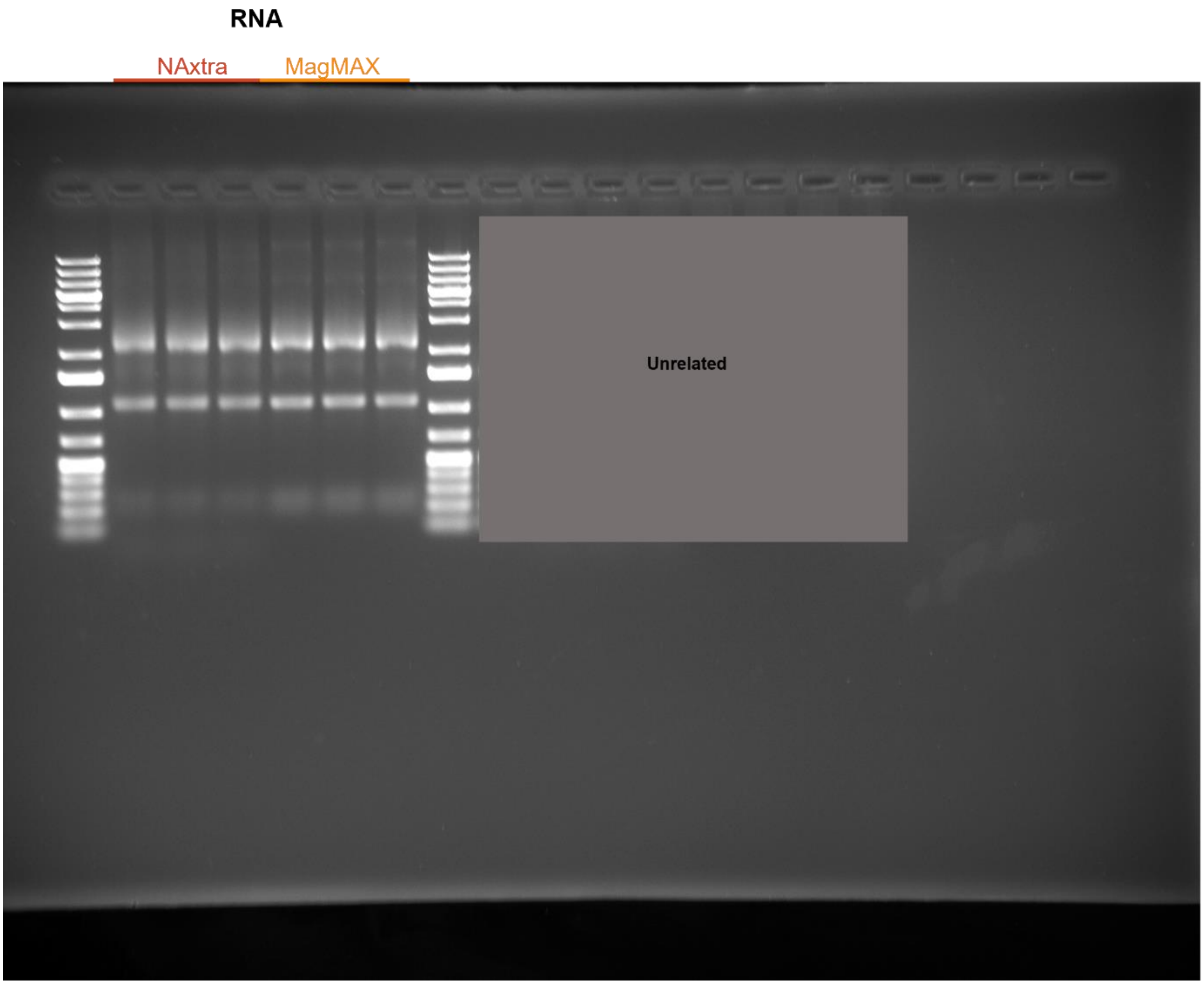

Supplementary figure 8. Original gel image for **Figure 7A**. RNA isolated from triplicates of 1 000 000 cells (HAP1) using the NAxtra-based method compared to the MagMAX *mir*Vana Total RNA isolation kit (Applied Biosystems) on KingFisher Duo Prime. Total RNA separated on a 1.2% agarose gel (5% eluate applied) with GeneRuler 1 kb Plus DNA Ladder (Thermo Scientific).
